# Bonobos respond prosocially toward members of other groups

**DOI:** 10.1038/s41598-017-15320-w

**Published:** 2017-11-07

**Authors:** Jingzhi Tan, Dan Ariely, Brian Hare

**Affiliations:** 1 Department of Evolutionary Anthropology, Duke University, Durham, NC 27708 USA; 2 Fuqua School of Business, Duke University, Durham, NC 27708 USA; 3 Center for Cognitive Neuroscience, Duke University, Durham, NC 27708 USA

## Abstract

Modern humans live in an “exploded” network with unusually large circles of trust that form due to prosociality toward unfamiliar people (i.e. xenophilia). In a set of experiments we demonstrate that semi-free ranging bonobos (*Pan paniscus*) – both juveniles and young adults – also show spontaneous responses consistent with xenophilia. Bonobos voluntarily aided an unfamiliar, non-group member in obtaining food even when he/she did not make overt requests for help. Bonobos also showed evidence for involuntary, contagious yawning in response to videos of yawning conspecifics who were complete strangers. These experiments reveal that xenophilia in bonobos can be unselfish, proactive and automatic. They support the first impression hypothesis that suggests xenophilia can evolve through individual selection in social species whenever the benefits of building new bonds outweigh the costs. Xenophilia likely evolved in bonobos as the risk of intergroup aggression dissipated and the benefits of bonding between immigrating members increased. Our findings also mean the human potential for xenophilia is either evolutionarily shared or convergent with bonobos and not unique to our species as previously proposed.

## Introduction

Trust is fundamental to social life^1^. One hallmark of human societies is that they have an unusually wide circle of trust. This includes unfamiliar individuals that can be anything from distant acquaintances to anonymous strangers. Conflicts occur between rival groups, but modern humans manage to live in a global social network connected by trusting relationships between unrelated strangers^2^. Contemporary hunter-gatherers commonly engage in cooperative interactions among unfamiliar individuals, so did early *Homo sapiens* (e.g. flexible dispersal, high social fluidity, intergroup alliance and long-distance trade^3–9^). This extensive circle of trust provides enormous benefits by creating an interconnected and ever-growing market for information, goods and support^10–18^. Such interconnectedness has been suggested to allow for cumulative culture and large-scale cooperation, two cornerstones of humanity^19–22^.

The human potential for xenophilia or prosociality toward unfamiliar individuals seems critical then to our species success in encouraging cooperation and cultural exchange^23^. In absence of past experience with strangers, humans rely on signals of positivity in establishing trust^24–26^. When encountering a stranger of unknown group membership humans are capable of making these positive signals with a prosocial first move^27^. This is in contrast to a negative or xenophobic response, and it does *not* require a prosocial preference for the unfamiliar over the familiar, although such a preference can be considered the extreme expression of xenophilia. A pattern of human xenophilia is observed across cultures and early in development^28–30^. It can occur even when the xenophilic actors obtain no selfish benefits, have limited cognitive control and receive no signals for help from the recipient. This profile suggests that human xenophilia is in part driven by unselfish motivations and automatic processes^31,32^. However, it remains unclear to what extent this kind of xenophilia evolved once our lineage split with other apes.

One hypothesis proposes that human xenophilia was derived in our lineage, which is supported by the larger pattern of xenophobia in most primates – including chimpanzees^8,33–36^. It has been suggested that human xenophilia evolved from the conserved fear of strangers seen across primates as a result of unique human bonding mechanisms such as intermarriage^37,38^ and cultural institutions^39^. Others suggest human xenophilia evolved due to ultra-strong prosocial motivation produced by group selection^40,41^ or cooperative breeding^31^.

The first impression hypothesis suggests that xenophilia evolves in response to the benefits of new social partners. This hypothesis predicts that prosocial responses to strangers can evolve in any social species where the selfish benefits of bonding with new partners outweigh the costs^15,42^. For example, xenophilia can be favored when there is limited risk of xenophobic aggression^43,44^. In this case positive encounters with strangers can develop into repeated interactions^41,45–48^. Strangers will become attractive social partners since social networks can be expanded through the formation of low risk and low cost “weak ties”^10,42^. A core prediction of the first impression hypothesis is that social preferences for positive interactions with non-group members (e.g. xenophilia) and cognition should evolve to support the network expansion of individuals when it enhances inclusive fitness^49^.

Bonobos (*Pan paniscus*) provide a powerful test of this prediction of the first impression hypothesis. Bonobos have been proposed as a product of selection against aggression or “self-domestication” that was driven by reduced feeding competition^44,50^. Bonobos not only possess a syndrome of morphological and physiological traits associated with domestication, but also show less severe forms of aggression than chimpanzees^44^. Territorial patrols, infanticide, and lethal intergroup aggression have never been observed in bonobos^51,52^. Tension can rise during intergroup encounters, but escalation into physical aggression with injuries is uncommon^52,53^. Instead, affiliative behaviors such as grooming, traveling together and socio-sexual behaviors have often been seen during interactions between immigrants or neighboring groups^52,54–59^. Unlike chimpanzees in captivity, there are no reports of bonobos killing adults or infants as a result of transfers between groups (although male immigrants without mothers can become targets of female aggression)^44^. Most importantly, while both bonobos and chimpanzees are patrilocal, only bonobo immigrants are attractive social partners for resident males and females^60,61^. Unrelated, immigrating members in bonobo groups even form alliances, gain priority of access to food and achieve high social status^52,62–64^.

Experiments have demonstrated that physical interactions with unfamiliar conspecifics can be rewarding for bonobos. Instead of monopolizing food in their possession, bonobos unlocked a one-way door in order to physically interact and co-feed for the first time with an unfamiliar neighbor^42,65^ (but not with a familiar recipient^66,67^). They often opened a second door for another non-group member even if it meant being outnumbered by non-group members – something chimpanzees actively avoid^68^. In another experimental context bonobos also released an unfamiliar conspecific into a room with food that they themselves could not access. This meant bonobos helped non-group members even when they received no social reward^42^.

However, it is still unclear how similar the xenophilic tendencies of bonobos are to that seen in humans - which can be unselfish, proactive and automatic toward complete strangers^27^. While proactive or unsolicited prosociality toward group members has been experimentally demonstrated in some contexts in chimpanzees and other primates^69–73^, proactive food provisioning of unfamiliar individuals from other groups remains little studied. Previous tests with bonobos have demonstrated their tendency to share with unfamiliar recipients from a different social group when using explicit measures of prosociality^42^. This work even suggests the potential for proactive sharing in bonobos since help was not contingent on gestures made by recipients. However, we remain without a strong test of (1) proactive sharing, (2) with completely novel conspecifics and (3) involuntary or implicit measures of social preferences commonly used in human research. We conducted a series of experiments to test the first impression hypothesis that meet these methodological challenges.

We first examined whether bonobos voluntarily provisioned an unfamiliar conspecific from a neighboring group who was unable to use overt signals to indicate their desire for help (i.e. since overt requests for help were prevented, aid was considered proactive^69,74^). We then tested whether bonobos had an involuntary contagious yawning response to complete strangers. Contagious yawning has been used by many as an implicit measure of social preference in various primates and non-primate species since it is under involuntary control^75–81^, although see^82,83^. Regardless of its exact proximate mechanism^84^, contagious yawning has been positively associated with social rapport in a variety of animals including humans and bonobos^85–87^. This includes work showing that yawn contagion in xenophobic chimpanzees is made in response to in-group but not out-group conspecifics^77^. These findings make contagious yawning a useful implicit measure of positive social preference. We therefore tested the first impression hypothesis by examining how bonobos help and contagiously yawn in response to unfamiliar conspecifics. The first impression hypothesis predicts that bonobos will proactively provision food to unfamiliar, non-group members and will contagiously yawn in response to complete strangers.

## Results

### Experiment 1

Bonobos (*n* = 16) could provide out-of-reach food to an unfamiliar recipient from another social group. The subjects and the recipients had never co-resided in the same enclosure and had never been paired in previous experiments^42,65^. They were from neighboring groups separated by fence, which allowed no physical interaction, but occasional visual and vocal contact – a context that promotes highly xenophobic responses in captive chimpanzees^88^.

**Figure 1:**
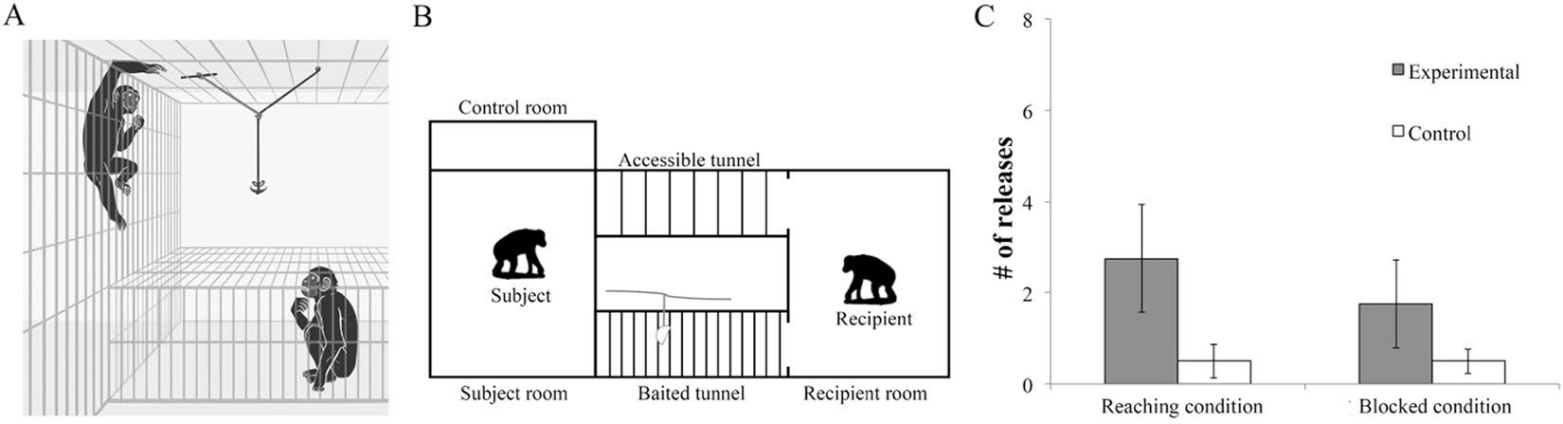
In experiment 1, bonobos could release a pin and cause high-hanging fruits to drop within the reach of an unfamiliar bonobo from neighboring groups (**A**). In the reaching condition, the recipient could pass his/her arm through the wide bars of the baited tunnel to signal for desire; in the blocked condition, such signals were blocked by the narrow bars (**B**). They released the apparatus more in the experimental condition than in the control condition, regardless of whether the recipient could signal for desire or not (**C**). Error bars show standard errors.

Experimenters tied a piece of apple to a rope hanging above the ceiling (Fig. 1). A wooden pin held the rope to the ceiling, and the food dropped once the pin was removed. We placed the rope above a mesh tunnel (the baited tunnel; Fig. 1) where the recipient could enter but could not reach the high-hanging food unless the subject, who was in a room adjacent to the tunnel, removed the pin and released the food. The subjects could never reach the food or the rope, and they demonstrated an understanding of this prior to the test (see Methods). However, they could release the pin for the recipient. This aid was designed to come at a cost to the subjects. They always started a trial on the opposite end of the subject room, where they could play with a desirable toy^89^. Subjects had to forego playing with the toy while paying the energetic cost of traveling across the room, climbing two meters, and suspending themselves by one arm in order to remove the pin for the recipient (Fig. 1). Releasing the pin gave no immediate, selfish benefit since 1) a physical social interaction was not possible between the separate rooms and 2) reciprocity was precluded by testing bonobos from different groups who could not interact outside the experiment. Also, the bonobos’ roles were never reversed within the experiment itself.

In the test phase, subjects could provision food in the *experimental condition* where the recipient could enter the tunnel below the food, but in the *control condition* food provisioning was not possible even if subjects released the pin since the recipient was in another adjacent room (the control room) with no access to the baited tunnel (See Fig. 1). In the experimental condition, we also manipulated the possibility for the recipient to use gestural signals. Half of subjects were randomly assigned to the *reaching condition* where the mesh of the baited tunnel was wide enough to allow the recipient to extend an arm through the bars and use a requesting gesture. The rest of the subjects were in the *blocked condition* where the mesh was too narrow for an arm to pass. This means that in neither condition could the recipient actually reach the high-hanging fruit and only in the reaching condition could recipient overtly signal to the subject his/her desire by extending the entire arm – the gesture bonobos typically use to request attention and food from others^90^.

Subjects provisioned food to the recipient by releasing the pin in the experimental condition more often than in the control (*n* = 16, *t* = 2.874, *p* = 0.012, paired t-test). We also observed food-provisioning that is consistent with criteria for proactive prosociality. First, when the recipient’s possibility for signaling with overt gestures was blocked subjects provisioned as often as they did when the gesturing was not blocked (food-provisioning tendency was operationalized as the difference in the number of trials a subject released the pin between the experimental and the control conditions, *n* = 16, *t* = −0.812, *p* = 0.431, independent t-test). Second, we coded whether the recipient actually signaled to the subject in each trial, because it is possible that in the reaching condition the recipient might not make any gesture or in the blocked condition the recipient might attempt more subtle gestures like pointing with fingers (see Methods for operational definitions and reliability). On average signaling behaviors were observed in 36.7 ± 27.2% of all experimental trials. Subjects’ food provisioning was not affected by whether the recipient made signaling behaviors or not (*n* = 14, *t* = 0.049, *p* = 0.962, paired t-test, two subjects could not be included in this analysis because their recipients showed no variance of signaling behavior by either always or never making any gesture). Third, our testing rooms separated the subject and the recipient with narrow mesh. This made it impossible for the recipient to harass or solicit the subject for help^91–94^. For example, the recipient could have shaken the mesh between the subject room and the baited tunnel to capture the subject’s attention, but this only occurred in two trials. Hence, there is little evidence that food provisioning was affected by the recipient’s attention-getting behavior.

A trial-by-trial analyses via generalized linear mixed model (GLMM) corroborated our more conventional analysis. We constructed a full model with whether the pin was released in a trial as a binary outcome variable (i.e. following a binomial distribution), with condition (i.e. experimental vs. control), trial number (i.e. 1–8), signaling possibility (i.e. reaching vs. blocked), a condition × trial number interaction and a condition × signal possibility interaction as fixed factors, with age as covariate, while accounting for the effect of repeated measures within subjects^95^. We first compared the full model with a null model that included only age, the intercept and the random effect via likelihood ratio tests, and we found a significant difference between the models (*χ* ^2^ = 47.78, *df* = 5, *p* < 0.001). Likelihood ratio tests of the full model revealed that the main effects of condition and trial number were significant, but their interaction was not (condition: *χ* ^2^ = 4.98, *df* = 1, *p* = 0.03; trial number: *χ* ^2^ = 11.15, *df* = 1, *p* < 0.001; condition × trial number: *χ* ^2^ = 0.64, *df* = 1, *p* = 0.42). Subjects released more often in the experimental condition, and their tendencies to release declined in *both* conditions at equal rate as the trial number increased (see also Melis *et al*.^96^). There was no significant effect of age, signaling possibility or the condition × signaling possibility interaction (age: *χ* ^2^ = 1.51, *df* = 1, *p* = 0.22; signaling possibility: *χ* ^2^ = 0.19, *df* = 1, *p = *0.66; condition × signaling possibility: *χ* ^2^ = 2.06, *df* = 1, *p* = 0.15). GLMM analysis of trials in the experimental condition further revealed that neither the signaling possibility nor the actual signaling behavior of the recipient had any effect on the subjects’ release behavior (signaling possibility: *χ* ^2^ = 1.24, *df* = 1, *p* = 0.27; signaling behavior: *χ* ^2^ = 0.02, *df* = 1, *p* = 0.89, see Supplementary Materials for more details of this GLMM analysis).

The aid that bonobos provided to the unfamiliar recipient also cannot be explained by social facilitation because the recipient was always in a room adjacent to the subject room in all conditions. It also cannot be accounted for by a lack of inhibitory control since all subjects passed an inhibition pretest (see Methods). Since recipients were free to move closer or farther away from the out of reach food, we examined whether their proximity affected the recipients helping. Subjects also did not release the pin more when the recipient had entered the tunnel below the rope with food during the experimental trials (*n* = 9, *t* = 1.744, *p* = 0.119, paired t-test, seven subjects were excluded from this analysis as the recipient either always entered or never entered into the tunnel below the rope and food). Trial-by-trial analysis via GLMM confirmed that the recipient’s entry into this tunnel had no effect on food provisioning (*χ* ^2^ = 0.61, *df* = 1, *p* = 0.43, see Supplementary Materials for more details of this GLMM analysis). This makes it difficult to attribute the subjects helping behavior to local enhancement since food provisioning did not increase when the recipient was near the apparatus. Although bonobos do seek opportunities to engage in physical interactions with individual from outside their own group^42^, it is unlikely that the observed helping was intended to bring the recipient into closer proximity. Releasing the food did not allow the recipient to come any closer while releasing the pin actually required that subjects moved *away* from the recipient. Finally, the release rate in the experimental condition was 28.1 ± 9%. This might be construed as relatively infrequent. However, the release rate in control trials was low 6.3 ± 2.8%. This suggests instead that the energetic and opportunity costs of releasing the apparatus were significant. This 20% increase in release rate between the two conditions is also similar to levels of helping observed in previous experiments with bonobos and chimpanzees, despite the higher baselines in previous experiments^42,91,96^. Future research should systematically manipulate the cost to the aid.

As female bonobos are not dominated by males but are the immigrating sex, xenophilia might be predicted to be stronger in our experimental pairs including females. However, subjects’ food provisioning tendency was not affected by the sex of the subject or the recipient (*n* = 16, *F* = 0.263, *p* = 0.85, one-way ANOVA). This may in part be that our experimental manipulation does not closely approximate the context in which immigration occurs in the wild. Our sample size is also limited for this type of analysis preventing us from including this variable in the above GLMM analysis (the model failed to converge, probably due to too many predictors for the current sample size).

Taken together, experiment 1 shows that bonobo xenophilia can be proactive and unselfish: they provisioned food to an unfamiliar recipient even when the recipient did not overtly signal for help and when there was no selfish benefit. This form of xenophilia was expressed between pairs of unfamiliar bonobos from neighboring groups who have never stayed in the same enclosure or physically interacted. It is unlikely they formed any type of social relationship beyond recognizing each other as members of another group. This is similar to what most primates experience in naturally occurring intergroup interactions – although it is likely these different groups of captive bonobos would have more exposure to each other than wild bonobos. In experiment 2 and 3, we further measured bonobo xenophilia when familiarity was at the lowest possible level: the first encounter between complete strangers.

### Experiment 2

**Figure 2:**
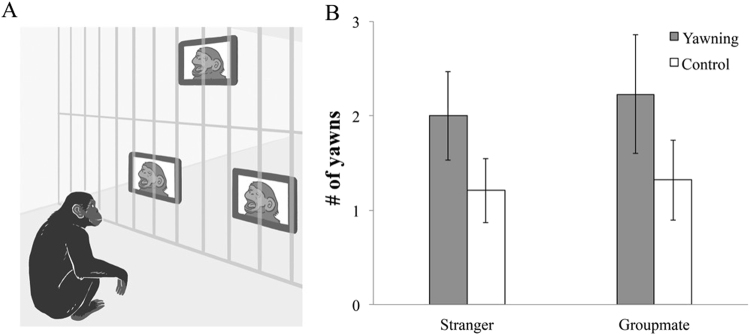
In experiment 2, subjects watched yawning and control videos of conspecific models that were either group members or complete strangers whom they have never met before (**A**). Subjects overall yawned more often when watching the yawning videos than the control ones (**B**). Error bars show standard errors.

Previous work with bonobos shows a consistent pattern of xenophilia in their explicit choices. Involuntary measures are thought to reflect emotional responses and unconscious bias in humans^97^. Here we investigated whether bonobos (*n* = 25) would involuntarily yawn contagiously in response to complete strangers. We also measured their yawn contagion with groupmates, which allows us to qualitatively compare our results to a previous chimpanzee study^77^. We showed bonobos different sequences of videos based on a 2 × 2 design with yawning and group membership as within-subject factors (Fig. 2, see Methods). This yielded four different types of video sequences (stranger-yawning, stranger-control, groupmate-yawning, groupmate-control). Each sequence consisted of 12 short clips played in a loop for 10 minutes. In a yawn sequence, each clip showed a complete yawn from a model bonobo; in a control sequence, each clip showed a neutral face of the same individuals from the yawn videos. Stranger models were three males and three females from the Columbus Zoo in the United States. Having lived on a different continent their entire lives we knew the subjects had never seen or met these individuals before. Groupmate models were age- and sex-matched as closely as possible and all lived in the subject’s group at the Lola ya Bonobo sanctuary, Democratic Republic of Congo.

Subjects yawned more often while watching a sequence with yawning than when watching a control sequence (*F*_1,20_ = 9.023, *p* = 0.049, 2 × 2 repeated measures ANOVA). No significant effects of group membership or interaction between yawning and group membership were detected (Fig. 2B). When we analyzed the data using GLMM, the results remained the same. We constructed a full model with how many times a subject yawned while she was watching a sequence as the outcome variable following a Poisson distribution, with condition (i.e. experimental vs. control), group membership (i.e. strangers vs. groupmates), age, a condition × group membership interaction and a condition × age interaction as fixed effects, while accounting for the effect of repeated measures within subjects. We first compared the full model with a null model that included only age, the intercept and the random effect via likelihood ratio tests, and we found a significant difference between the models (*χ* ^2^ = 11.21, *df* = 4, *p* = 0.024). Likelihood ratio test revealed that the main effect of condition was significant, but no other term was (condition: *χ* ^2^ = 4.07, *df* = 1, *p* = 0.04; group membership: *χ* ^2^ = 0.06, *df* = 1, *p* = 0.81; age: *χ* ^2^ = 0.29, *df* = 1, *p* = 0.59; condition × group membership: *χ* ^2^ = 0.01, *df* = 1, *p* = 0.93; condition × age: *χ* ^2^ = 1.22, *df* = 1, *p* = 0.27). This GLMM analysis also revealed no age effect on yawn contagion in our sample^98^.

Experiment 2 replicates previous demonstrations of contagious yawning in bonobos^87^. It also provides the first evidence of bonobo xenophilia using an *implicit* measure, given that yawning is involuntary and the strength of its contagion increases as social rapport increases^78^. Our results suggest that bonobo xenophilia is in part driven by an implicit, automatic process, which might modulate their explicit helping. Our results also extend bonobo xenophilia to complete strangers. We measured *true* first impressions since our subjects had never met the strangers until they watched the videos - but in this first exposure, they reflexively showed a positive response.

It is unlikely that our subjects perceived the exposed canines in the yawning videos as a threat or a stress response, since 1) bonobos do not have facial expressions that act as formal signals of dominance^99^, 2) the contagiousness of a yawn in bonobos was previously observed to be highest in response to individuals posing the least threat to subjects^87^, 3) all video clips were recorded when the models were resting and 4) contagious yawning appears to be unrelated to stress responses in great apes^75^.

Although not the primary test of our predictions, it is interesting to note that stranger yawns were at least as contagious as those of groupmates. Because the contagiousness of bonobo yawns is an indicator of social rapport^87^, this result suggests that the strength of prosociality toward strangers and that toward groupmates is similar. This is in line with a previous experiment showing bonobos help groupmates and unfamiliar non-groupmates at an equal rate^42^ as well as with reports of bonobos welcoming newly transferred individuals like they were group members^54,56,57,59^. It is also different from chimpanzees tested in a similar experimental set up that only yawed contagiously in response to videos of groupmate yawning but not strangers^77^ (see Supplementary Materials for additional analyses of subgroups of our data that best match this chimpanzee study).

### Experiment 3

One low-level interpretation of the results from experiment 2 is that subjects somehow failed to recognize the group membership of the individuals depicted in the videos. While unlikely, we further tested this in a control experiment (*n* = 22) in which subjects could choose to receive a small, immediate reward or to watch videos of conspecifics while waiting for a large, delayed reward (see Methods)^100,101^. In the groupmate condition videos of groupmates were shown during the delay while in the stranger condition strangers from the Columbus Zoo were shown during the delay. Videos were formatted and presented in the same way as in experiment 2. Each video showed a head shot of a bonobo without facial expression. Given their xenophilic preferences, we predicted the bonobos would be more willing to wait out the delay while watching strangers than groupmates.

We found that on the first day subjects chose to wait more often when they were shown the stranger clips during the delay but by the second session (run on another day) subjects, now familiar with the stranger’s photos, no longer showed this preference (day 1: *n* = 22, *t* = 2.435, p = 0.024; day 2: *n* = 20, *t* = 0.325, p = 0.749, independent t-test; overall: *n* = 20, *t* = 1.602, *p* = 0.126, paired t-test). Subjects’ first-day preference for the stranger clips shows they do discriminated between strangers and groupmates depicted with video. While this result alone cannot show whether subjects attributed positive or negative valence to the stranger stimuli, it is consistent with the xenophilic responses shown in experiment 1–2 and previous studies^42^.

## Discussion

In strong support of the first impression hypothesis bonobos proactively provisioned out-of-reach food to an unfamiliar non-group member and showed involuntary, contagious responses to the yawns of complete strangers. The aid that bonobos explicitly provided the unfamiliar recipient in obtaining food is consistent with common definitions of proactive prosociality^69,74^, while contagious yawning suggests their xenophilia is not completely under voluntary control and is present even when there is zero familiarity. This bonobo pattern of xenophilia resembles contagious yawning and unconscious mimicry seen in humans more than chimpanzees^77,102,103^, as well as the heuristic-like response that drives human sharing with strangers in controlled experiments^27^. Like humans, bonobos proactively help unfamiliar conspecifics and their positive response is at least in part automatic.

Xenophilia in the current experiments was directed to conspecifics with various levels of familiarity, from neighbors with whom the subjects had never shared an enclosure to completely novel individuals. The xenophilia observed in this sanctuary sample is consistent with reports from a wide range of field sites and captive facilities showing that bonobos display affiliative behaviors toward acquaintances and new immigrants^52,54–57,104^. Our findings together with these observations do not support the alternative that our subjects have become xenophilic due to repeated testing^71,105^, due to their rearing history^106^, or due to the sanctuary environment that allowed visual and vocal contacts across group barriers^107^. Given that sanctuary bonobos are relatively risk-averse and indifferent to novel stimuli in non-social contexts^108,109^, a general attraction to novelty clearly cannot explain our findings. Finally, the social context at the sanctuary is highly similar to the experience of most wild primates that often see or hear neighboring groups but rarely physically interact with them due to the potential cost of aggression. Despite this, and unlike chimpanzees, bonobos in the wild and in our experiments appear to have evolved a xenophilic preference for this same type of stranger^52,110^.

It is difficult to explain the prosociality observed here as a result of harassment, reciprocity or a lack of inhibition since subjects could not physically interact with recipients, they had never been in the same group and pretests demonstrated subjects understood the experimental set-up (i.e. they passed self-regard pretests^111^). The physical setup and presence of a conspecific in the controls rule out mechanisms such as local enhancement or social facilitation. We are unaware of any evidence that bonobos can solicit the novel form of help tested here through non-gestural cues (e.g. subtle vocalizations, facial expressions or even situational cues^112^). This might be an interesting topic for future research, given bonobos are relatively sensitive to human social cues^113–115^. Moreover, based on our control the contagious yawning results cannot be attributed to an inability of subjects to discriminate strangers from groupmates in the videos.

According to the first impression hypothesis, xenophilia evolves when the benefits of bonding with new partners outweigh the costs. In the case of bonobos, strong female alliances and sexual selection against male aggression likely removed the threat of lethal intergroup aggression that drives chimpanzee xenophobia^44,116^. This might have turned a costly interaction into a highly beneficial one. Xenophilia was increasingly favored as strangers became more likely to turn into valuable long-term social partners. It is important for future studies to test other predictions of the first impression hypothesis. Future research will need to examine the role of age and sex as it relates to the strength of xenophilia in bonobos. For example our subjects were relatively young (4–18 years old), and within the age range (6–14 years old) that bonobos typically leave their natal groups in the wild^52^. Bonobos may show a different preference once they are past this age. Or the preferences of older adults may vary depending on the sex of the stranger. The first impression hypothesis predicts that, unfamiliar adult females will be preferred over adult males and that unfamiliar adult males may illicit xenophobic responses in some contexts (i.e. interactions between two strange adult males). It will be exciting to take this next step and understand which kind of strangers bonobos respond with xenophilia.

Another powerful test of this hypothesis will be quantitative comparisons between spontaneous responses toward strangers in bonobos and chimpanzees^110^. Despite a growing literature documenting bonobo xenophilia and chimpanzee xenophobia, most studies, including the current one, have focused on one species. This creates methodological variations across paradigms that prevent a more precise comparison between the two species^117^. Although in one current experiment (experiment 2) we made the design and the analysis as comparable to the chimpanzee study by Campbell *et al*.^77^ as possible, our comparison is still qualitative. Methods that allow for direct quantitative comparisons of both species are still needed^118^ (see Supplementary Materials). This comparison would be particularly powerful if it used eye tracking techniques to examine attention as it relates to yawning rates. It might be important to correct for higher levels of attention given to the yawns of strangers than those of groupmates.

While there is consensus that contagious yawning is involuntary, the nature of the mechanism driving this automatic response is still unclear^82–84^. Future research will be needed to understand if bonobo contagious yawning is an expression of some basic form of empathy^119^, a ‘social heuristic’^27^, or an oxytocin-vasopressin mediated response^120–122^. Regardless of the exact mechanism, our results suggest that bonobos have an involuntary positive response to complete strangers. As described for humans, bonobos seem predisposed to making a good “first impression” when interacting with a new social partner. For both humans and bonobos, many strong bonds likely start from positive encounters between unfamiliar adults catalyzed by xenophilia^3,8,52,56,123^. However, our results also suggest how xenophilia is constrained in both species in different ways. For example, the strong xenophobic reaction that humans display toward strangers from different cultural mediated outgroups severely limits human intergroup prosociality^8,124–126^. Likewise, while bonobos potentially even prefer an unfamiliar conspecific from another group over their own groupmate, they are probably much less flexible in terms of the contexts and the consistency with which they will provide aid (e.g. food-provisioning to strangers did not occur when the cost became considerably high^42^).

The most exciting puzzle for the future will be determining why humans evolved the potential for trusting relationships with strangers in a wider variety of contexts – allowing rapid diffusion of information and reciprocal between-group cooperation^5,7,10,17,49,123^. This will require uncovering whether some forms of xenophilia are shared or convergent between humans and bonobos^23^. Regardless of the answer, bonobo networking has much to teach us about the origins of the human network we all rely upon.

## Methods

### Ethical Note

The current experiments were conducted in Lola ya Bonobo sanctuary, Kinshasa, Democratic Republic of Congo. Most subjects were orphans from bushmeat trade and were living in social groups. In the daytime, they had access to heavily forested outdoor enclosure and at night they slept in multi-room dormitories. A previous comparison found no differences in psychological health between orphan and mother-reared bonobos living the sanctuary^106^. The experiments were conducted in their sleeping dormitory and before the morning meal to maximize their motivation for food. Subjects were never food-/water-deprived. They could leave the testing rooms any time by sitting next to the exits. The video collection process took place in Columbus Zoo, Ohio, USA. The current research has been approved by the IACUC committee of Duke University (#A078–08–03), Columbus Zoo and Lola ya Bonobo sanctuary. The current research was carried out in accordance with the guidelines and regulations of these three institutions and the countries in which the work was conducted.

### Experiment 1

Sixteen bonobos (8 F:8 M, mean age = 9.2, range from 6 to 15) participated in experiment 1. The sexes of the subject and the recipient were counterbalanced between pairs (see Table S1).

The setup of the experiment consisted of a subject room and a recipient room connected by two parallel tunnels (the baited tunnel and the accessible tunnel, Fig. 1). In addition, there was a control room adjacent to the subject room where the recipient was placed in the control condition (see below). The testing rooms had a meshed ceiling with wide bars where the subject was able to put his/her arms through to reach for items. However, when a bonobo was inside the tunnels the ceiling was out of his/her reach. On top of the baited tunnel was the testing apparatus hanging from the ceiling. The apparatus comprised of a horizontal rope and a vertical rope. One end of the horizontal rope was fastened to the ceiling at the midpoint above the baited tunnel. We created a loop at the other end and placed a wooden pin through it. Because the pin was just long enough to bridge two adjacent bars on the ceiling, this end of the horizontal rope could be hung on the ceiling but could be easily released by twisting, removing or breaking the pin. Subjects could only reach the pin and could never pull the rope into the subject room. The vertical rope was attached to the midpoint of the horizontal rope and was hanging a food piece just above the baited tunnel. Before the apparatus was released, the vertical rope was out of the subject’s and the recipient’s reach. Once the horizontal rope was released, the food would drop right on the baited tunnel so the recipient inside could obtain the food (see Supplementary Materials).

Before the test phase, we conducted three pre-tests to make sure that 1) the subjects understood the release mechanism of the apparatus, 2) they understood that, once the apparatus was released, the food could only be obtained from the baited tunnel, and 3) removing the pin was not intrinsically motivating.

The experiment procedure consisted of four phases: self-regard pretest I, self-regard pretest II, no-food introduction and test. The self-regard pretests were conducted on one testing day and the last two phases on a subsequent day.

Self-regard pretest I: This was designed to show that the subjects understood the release mechanism of the apparatus. The apparatus was set up on the ceiling above the baited tunnel. In this phase the accessible tunnel was closed at both ends, while the baited tunnel was open to the subject room. The subject could climb up the subject room, put his/her arm through the mesh to release the apparatus and then enter the tunnel to obtain the food. The subject could proceed to the next phase if he/she successfully obtained food in 4 consecutive one-minute trials.

Self-regard pretest II: This phase was designed to demonstrate the subjects understood that the food piece could be obtained by a bonobo entering the baited tunnel from the recipient room. This phase was identical to self-regard pretest I except: 1) the accessible tunnel was open at both ends so that the subject could travel between the two rooms freely; 2) the baited tunnel was closed from the subject room while open from the recipient room so that he/she could enter the tunnel only from the recipient room. Therefore, the subject had to make a detour to obtain the reward after releasing the apparatus. Subjects passed this phase after they obtained food in 4 consecutive one-minute trials.

No-food introduction: This phase was designed to demonstrate that 1) the subject understood when the tunnels were closed, he/she could never obtain the food even if the apparatus was released, and 2) releasing the apparatus was not intrinsically motivating in itself. In this phase, both tunnels were closed from the subject room and the fruit piece was unobtainable even if subjects released the apparatus. To provide the subject an alternative activity, we attached a toy made of two PVC tubes at the other end of the subject room. To pass this phase, the subject needed to inhibit releasing the apparatus in 4 consecutive one-minute trials.

Test: This phase comprised of two conditions administered immediately after the no-food introduction. Identical to the no-food introduction, both tunnels were open from the recipient room but closed from the subject room. In the experimental condition, the recipient was in the recipient room. For eight subjects the baited tunnel had wide bars so that the recipient could signal desire by extending arms (a.k.a. the reaching condition); for the other eight subjects, the bars of the baited tunnel were narrow and the recipient could not signal desire overtly by extending arms (a.k.a. the blocked condition, Fig. 1B). In the control condition, the recipient was moved to the control room to control for the potential effect of social facilitation (i.e. an general increase of activity level caused by the mere presence of other individuals^42,96^). Each subject received 8 one-minute trials per condition (16 trials in total per subject) administered in a block design. The order of the conditions was counterbalanced between-subjects.

A *release* was coded when the subject dropped the food attached to the vertical rope by twisting, removing or breaking the pin holding the horizontal rope. We also coded several types of recipient’s behaviors in the experimental condition that might affect the subjects’ food-provisioning. *Signaling behavior* was when, before a release, the recipient 1) extended his/her arms, hands or fingers through the mesh of the baited tunnel toward the hanging food, and/or 2) attempted to reach the food from the top of the recipient room. *Attention-getting* was coded when, before a release, the recipient shook the mesh separating the subject room and the baited tunnel. *Entry* was recorded when the recipient went inside the baited tunnel during a given trial. Our experimental setup did not allow for the coding of facial expression or vocalizations.

A second coder blind to the testing condition and hypothesis coded 25% randomly selected trials. Inter-coder reliability was excellent (release: kappa = 1; signaling: kappa = 0.936; attention-getting: one disagreement among 32 trials, unable to calculate kappa; entry: kappa = 1). Data were analyzed using R Studio (version 0.99.892) and statistical packages *lme4* and *afex* ^127–129^.

### Experiment 2

A total of 25 bonobos (12 F:13 M, mean age = 9.4, range from 4 to 18) participated in experiment 2 (see Table S2). Each subject watched four video sequences, except four bonobos. Two of these four bonobos only watched the stranger stimuli because they were from a small group and did not have enough groupmates to produce the stimuli. For the last two bonobos, one only watched the groupmate stimuli and the other only watched the stranger stimuli due to experimenter error (see Supplementary Materials).

Each sequence consisted of 12 short clips from six adult models (3 F:3 M, two clips per bonobo). Each sequence lasted for approximately two minutes and was played in a loop for 10 minutes. Yawning and control clips were extracted from the same source videos. They were thus matched in playing order, video length, brightness, contrast, the model’s identity and body posture.

We created the video stimuli in.AVI format with a 720 × 540 resolution and presented them to the subjects with three 8-inch LCD monitors (NIX Pro-Series digital photo frame). These screens were synchronized and set up behind the same side of the mesh approximately 90 cm outside the testing room (Figs 2A and S2). In this way, subjects were exposed to the stimuli whenever they were looking at that side of the testing room.

Each subject participated in two testing days and watched two sequences of the same models on each day. To prevent bonobos habituating to the videos, the two testing days were at least 5 days apart. The order of conditions was counterbalanced between subjects. Before showing each sequence, there was a 5-minute arousal phase where an experimenter was tickling and feeding the subjects with 15 fruit pieces in order to keep them aroused. This was because 1) we conducted the experiment early in the morning when yawning might be at a high level^130^, and 2) each subject watched two sequences per day so that the stimuli in the first session might have a carry-over effect that last into the second one^117^. After the arousal phase, the sequence was played on the screens in the repeat mode for a total of 10 minutes.

We videotaped subjects’ behaviors during the 10-minute exposures to the stimuli. We coded a *yawn* when subjects made a slow gaping movement that was not in the context of feeding, vocalizing or playing. A second coder blind to the testing condition coded 20% randomly selected trials. Inter-coder reliability was excellent (*n* = 18, *r* = 0.981, *p* < 0.001, Spearman’s correlation).

### Experiment 3

Twenty-two bonobos (10 F:12 M, mean age = 9.9, range from 5 to 17) participated in experiment 3 (see Table S3). There were two conditions and subjects were tested in one condition per testing day (except that two subjects lost motivation after the first day, see Supplementary Materials). Each condition consisted of 10 test trials in a block design.

Subjects could choose between an instant option and a delay option that were approximately 1.5 meters apart. The instant option had two small pieces of apple that were immediately fed to the subjects if they chose it; the delay option had six pieces that, if chosen, were delivered to the subjects after a one-minute delay (based on Rosati *et al*.^100^). The delay option also had two 8-inch LCD monitors attached back-to-back and fixed to a bookstand with six apple dices in the front (see Fig. S3). During each delay, subjects could view a video clip showing a close-up shot of a bonobo model. The front screen served as a preview screen showing a static shot of the clip. The rear screen was playing the corresponding clip in the repeat mode. Once subjects chose the delay option, the experimenter would turn the rear screen to the front and show the clip for one minute.

In the stranger condition, there were 10 clips showing 10 bonobo models (5 F:5 M) from the Columbus Zoo. Subjects have never met or seen these bonobos, except for two who were also yawning models in experiment 2. In the groupmate condition, the clips were 10 bonobo models (5 F:5 M) from each subject’s own group. The models were all in relaxed state with no facial expression. The two sets of models were matched in regard to presentation order, sex and age group. Subjects have never seen any of these clips unless they chose to watch it during the test. All clips were formatted and displayed in the same way as in experiment 2.

In each day subjects started with a choice introduction phase designed to introduce the contingency of each option (based on Rosati *et al*.^100^, see Supplementary Materials) and then proceeded to the test phase. In each test trial subjects had 30 seconds to make a *choice* by placing their fingers or sitting down in front of the option. A second coder blind to the hypothesis and the testing conditions coded 28.5% randomly selected trials for subject’s choices (kappa = 1).

## Electronic supplementary material

Supplementary Materials

Table S1

Table S2

Table S3
