# Supplementary material for: Bonobos respond prosocially toward members of other groups
Source: Sci Rep. 2017 Nov 7;7:14733. doi: 10.1038/s41598-017-15320-w (PMC5676687; doi:10.1038/s41598-017-15320-w)
Supplement: Supplementary file 1 — Supplementary Materials [file 41598_2017_15320_MOESM1_ESM.doc]

**Supplementary Materials**

**Bonobos respond prosocially toward members of other groups**

Jingzhi Tan^1*^, Dan Ariely^2,3^, Brian Hare^1,3^

^1^ Department of Evolutionary Anthropology, Duke University, Durham, NC, USA, 27708

^2^ Fuqua School of Business, Duke University, Durham, NC, USA, 27708

^3^ Center for Cognitive Neuroscience, Duke University, Durham, NC, USA, 27708

^*^ corresponding author: [jingzhi.tan@duke.edu](mailto:jingzhi.tan@duke.edu)
Box 90383, 130 Science Drive, Durham, NC, 27708, USA

**Experiment 1**

**Supplementary methods**

Table S1 listed information and results of each subject in this experiment.

The distance between the top of the baited tunnel and the ceiling where the apparatus was fastened was 1.2m. The horizontal rope was 70cm in length. In the reaching condition, the vertical rope is 20cm in length; in the blocked condition, it is 1m in length. The vertical rope was tied to the mid-point of the horizontal rope. In the reaching condition, the bars of the baited tunnel were 10cm apart, allowing the recipient’s arm to pass through; in the blocked condition, the bars were 5cm apart, blocking any arm-reaching.

**Supplementary analyses and results**

We did not include a condition × age interaction into our GLMM model of the experimental condition and the control, because all of our subjects and recipients were within the age range of 6-15 – the typical time for bonobo immigration (Furuichi 2011). Therefore, the first impression hypothesis has no prediction of an age effect on xenophilia within this range. However, future research should certainly expand into older samples.

For GLMM analyses of the effects of signaling possibility, signaling behavior and social enhancement in the experimental trials, we constructed the full models with whether the pin was released in an experimental trial as a binary outcome variable (i.e. following binomial distribution), with age as a control covariate, with trial number (i.e. 1-8) plus one of the three variables (signaling possibility, signaling behavior or the recipient’s entry into the accessible tunnel) as fixed factors, and with subjects as random intercept. We first compared these GLMM full models to the null model including only age and the random intercept. All three full-null tests yielded significant differences, warranting further analyses of fixed effects (Signaling possibility model: *χ^2^* = 7.62, *df* = 2, *p* = 0.02; signaling behavior model: *χ^2^* = 6.40, *df* = 2, *p* = 0.04; entry model: *χ^2^* = 6.98, *df* = 1, *p* = 0.03). Subsequent likelihood ratio tests revealed no significant effect of signaling possibility, signaling behavior or entry (as reported in the main text).

Finally, the observed xenophilia is unlikely an artifact of rearing history and housing condition. Our results from a sample of orphans are consistent with reports from a wide range of field sites and captive facilities showing bonobos are peaceful and friendly toward strangers (Wilson et al. 2014, Furuichi 2011, Holt & van Elsacke 1990, Gold 2001, Pfalzer & Ehret 1995, Idani 1991, Hohmann 2001). Likewise, rearing history and housing conditions have not altered the xenophobic nature of chimpanzees (Brent 2001, Campbell & de Waal 2011).

**Experiment 2**

**Supplementary methods**

Table S2 listed information and results of each subject in this experiment.

Three female bonobos (Lukuru, Kananga, Lisala) only watched stimuli of strangers and one male bonobo (Kasongo) only finished the groupmate condition. Lukuru and Kananga lived a small group that did not have enough individuals to produce the groupmate stimuli. The groupmate conditions of Lisala and the stranger conditions of Kasongo were aborted because of experimenter error.

For the subjects who were also model bonobos in the clips, their clips were removed from the groupmate sequence (i.e. they watched 10-clip sequences in the groupmate conditions), but they were still exposed for the same amount of time (10 mins) as in the stranger conditions. The order of clips in each sequence was fixed: three male clips, three female clips, three males, and three females. Each clip lasted for 6-16 seconds and started with a 2-second attention-getter (i.e. a bright yellow screen and a ringing sound). Other than the initial ringing sound, the clips were silent. In each clip there was only one bonobo.

Two screens were placed on testing tables 80 cm above the ground and 1-1.5 m between each other. The third screen was mounted on a metal rack that stood 1.6 m above the ground and in the midpoint of the two lower screens. The screens were controlled by a single remote. In this way we created a “wall” of synchronized screens. Experimenter 1 (E1) and 2 (E2) each held a JVC or a SONY Handycam to videotape the experiment from two different angles. E1 always held the primary camera to videotape from the side, while E2 videotaped from the back of the room or the back of the screens. Coding was mainly based on E1’s recording but we referred to E2’s recording when subject’s face cannot be seen from E1’s camera.

Subjects were tested alone (n = 18), with an infant (n = 4) or with an adult companion (n = 3).

In our GLMM analyses, we only included a condition × age interaction due to the literature on the development of contagious yawning in great apes (e.g. Madsen et al. 2013). We did not include either a group membership × age interaction or a condition × group membership × age three-way interaction. Like in Experiment 1, this is because all of our subjects and recipients were within the age range of 6-15 – the typical time for bonobo immigration (Furuichi 2011). Therefore, the first impression hypothesis has no prediction of an age effect on xenophilia within this range. However, future research should certainly expand into older samples.

Yawning *latency* referred to the time between the start of the watching phase and the onset of the first yawn observed. Inter-coder reliability of latency was excellent (*n* = 12, *r* = 1.000, *p* < 0.001, Spearman’s correlation, two-tailed).

**Supplementary results**

Latency analysis showed that subjects yawned neither earlier nor later in the experimental condition than in the control (stranger: *N* = 10 (no ties), *Z* = -1.172, *p* = 0.241; groupmate: *N* = 6 (no ties), *Z* = -0.524, *p* = 0.600, Wilcoxon test, two-tailed).

We analyzed a subgroup of subjects that were tested first in the groupmate conditions and then in the stranger conditions, because this was the testing order used by Campbell and de Waal (2011) to find that chimpanzees only yawn contagiously with groupmates. We again found that subjects showed yawn contagion in the stranger conditions but not in the groupmate conditions (stranger: *N* = 11 (four ties), *Z* = -1.983, *p* = 0.047; groupmate: *N* = 11 (three ties), *Z* = -1.198, *p* = 0.231, Wilcoxon test, two-tailed). The strength of contagion did not differ between stranger and groupmate conditions (*N* = 11 (three ties), *Z* = -0.422, *p* = 0.673, Wilcoxon test, two-tailed).

We also analyzed a subgroup of subjects that were at least 10 years old, because Campbell and de Waal (2011) only used chimpanzees in the age range. We again found that subjects showed yawn contagion in the stranger conditions but not in the groupmate conditions (stranger: *N* = 12 (three ties), *Z* = -2.461, *p* = 0.014; groupmate: *N* = 11 (four ties), *Z* = -0.171, *p* = 0.864, Wilcoxon test, two-tailed). The strength of contagion did not differ between stranger and groupmate conditions (*N* = 11 (two ties), *Z* = -1.429, *p* = 0.153, Wilcoxon test, two-tailed).

Finally, whether subjects were tested alone or in pairs had no effect on the yawn contagion with strangers or groupmates (stranger: *N* = 24, *U* = 55, *p* = 0.570; groupmate: *N* = 22, *U* = 52.5, *p* = 0.808, Mann-Whitney test, two-tailed). Whether subjects were male or female had no effect either (stranger: *N* = 24, *U* = 66, *p* = 0.743; groupmate: *N* = 22, *U* = 58.5, *p* = 0.920, Mann-Whitney test, two-tailed). We were unable to analyze the effect of the models’ sex because both male and female clips were compiled into each sequence.

**Experiment 3**

**Supplementary methods**

Table S3 listed information and results of each subject in this experiment.

Two male bonobos lost motivation after finishing only the groupmate condition in Day 1 (a loss of motivation is defined as a refusal to eat food in three consecutive trials during the choice introduction phase). Another female lost motivation during the introduction phase and was dropped from all analyses. Fourteen subjects of experiment 3 have participated in Experiment 2.

Each clip lasted for four seconds and began with a bright yellow fixation screen and a ring sound to capture attention. The rest of the clips were silent. All clips were formatted to .AVI and 720 × 540 in resolution. We used 8-inch screens (NIX™ Pro-Series 8” digital photo frames) to play the stimuli (same as in experiment 2). For the subject who was also included in the stimuli as a model, we replaced its clip with that of another sex-matched bonobos from its age cohort.

Male stimuli included four adults and one infant, and female stimuli included three adults and two juveniles. The presentation order of the clips in each sequence was always the same, starting with an adult male and alternating between male and female clips each trial. One additional clip of an 11th groupmate model was created for the choice introduction phase.

We used two sliding tables to present the options in front of the mesh of the testing room. The distance between the two options was approximately 1.5 meters (with the exception that for Malayika the distance was only 0.9 meter due to the limited meshed area in her testing room).

Choice introduction phase: This phase introduced the contingency of the instant and the delay option to the subjects. There were two sliding tables but only one option in each trial. The experimenter (E) first centered the subject, placed the apparatus on the designated table, showed the food pieces to the subject, placed the food in front of the apparatus, and pushed both tables forward. In this 10-trial phase, E presented each option to the subjects five times in a counterbalanced order and they could have 30 seconds to “choose” the option. All five trials with the delay option were playing the same extra clip of the 11th groupmate model.

Test phase: Immediately after the choice introduction phase, the subjects received 10 test trials. These trials were identical to the introduction except that 1) both options were present and 2) the models in the clips were different in every trial. The locations of the options were counterbalanced both within and between subjects.

After E pushed the sliding tables forward, a *choice* was coded when the subjects 1) placed their fingers through the mesh in front of the table with an option, or 2) moved their bodies from the center to sit in front of that table. A trial was re-run if 1) the subjects did not made a choice within 30 seconds, 2) they were distracted when E was baiting the rewards, or 3) they made a choice before E pushed the tables.

**Supplementary results and discussion**

We replicated the findings of Rosati and colleagues (2007, 2013) by showing that subjects preferred the delay option to the instant option in both conditions (stranger: *N* = 20, *Z* = 2.447, *p* = 0.014; groupmate: *N* = 22, *Z* = 2.021, *p* = 0.043, Wilcoxon test). Future studies that increase the delay might be able to detect a stronger xenophilic effect (Rosati et al. 2007). Finally, we also found no effect of subject sex (stranger: *N* = 20, *U* = 40.5, *p* = 0.461; groupmate: *N* = 22, *U* = 34, *p* = 0.068, Mann-Whitney test, two-tailed). We were not able to analyze the effect of the models’ sex because the clips were in a fixed order without counter-balancing the models’ sex.

**Supplementary references**

Amici F, Aureli F, Call J (2014) Response facilitation in the four great apes: is there a role for empathy? *Primates*. 55:113-118.

Brent L (2001) *The care and management of captive chimpanzees* (American Society of Primatologists, San Antonio, TX.).

Campbell MW, de Waal FBM (2011) Ingroup-outgroup bias in contagious yawning by chimpanzees supports link to empathy. *PLoS One* 6(4):e18283.

de Waal FBM (1988) The Communicative Repertoire of Captive Bonobos (Pan paniscus), Compared to That of Chimpanzees. *Behaviour* 106(3):183–251.

Demuru E, Palagi E (2012) In Bonobos Yawn Contagion Is Higher among Kin and Friends. *PLoS One* 7(11).

Furuichi T (2009) Factors underlying party size differences between chimpanzees and bonobos: a review and hypotheses for future study. *Primates* 50(3):197–209.

Gold K (2001) Group formation in captive bonobos: sex as a bonding strategy. *The Apes: Challenges for 21st Century* (Brookfield Zoo, Brookfield), pp 90–93.

Hohmann G (2001) Association and Social Interactions Between Strangers and Residents in Bonobos (Pan paniscus). *Primates* 42:91–99.

Holt T, van Elsacke L (1990) A preliminary study on the social behaviour of captive bonobos. *Acta Zool Pathol Antverp* 81:31–39.

Idani G (1991) Social Relationships between Immigrant and Resident Bonobo *(Pan paniscus)* Females at Wamba. *Folia Primatol* 57(2):83–95.

Madsen, E.A., Persson, T., Sayehli, S., Lenninger, S., and Sonesson, G. (2013). Chimpanzees Show a Developmental Increase in Susceptibility to Contagious Yawning: A Test of the Effect of Ontogeny and Emotional Closeness on Yawn Contagion. *PLoS One* 8, e76266.

Pfalzer S, Ehret GG, Ulm U (1995) Social integration of a bonobo mother and her dependent daughter into an unfamiliar group. *Primates* 36(3):349–360.

Rosati AG, Stevens JR, Hare B, Hauser MD (2007) The evolutionary origins of human patience: temporal preferences in chimpanzees, bonobos, and human adults. *Curr Biol* 17(19):1663–8.

Rosati AG, Hare B (2013) Chimpanzees and Bonobos Exhibit Emotional Responses to Decision Outcomes. *PLoS One* 8(5):e63058.

Tan J, Hare B (2013) Bonobos Share with Strangers. *PLoS One* 8(1):1–11.

Wilson ML, Hauser MD, Wrangham RW (2001) Does participation in intergroup conflict depend on numerical assessment, range location, or rank for wild chimpanzees? *Anim Behav* 61(6):1203–1216.

**Supplementary figures**

**
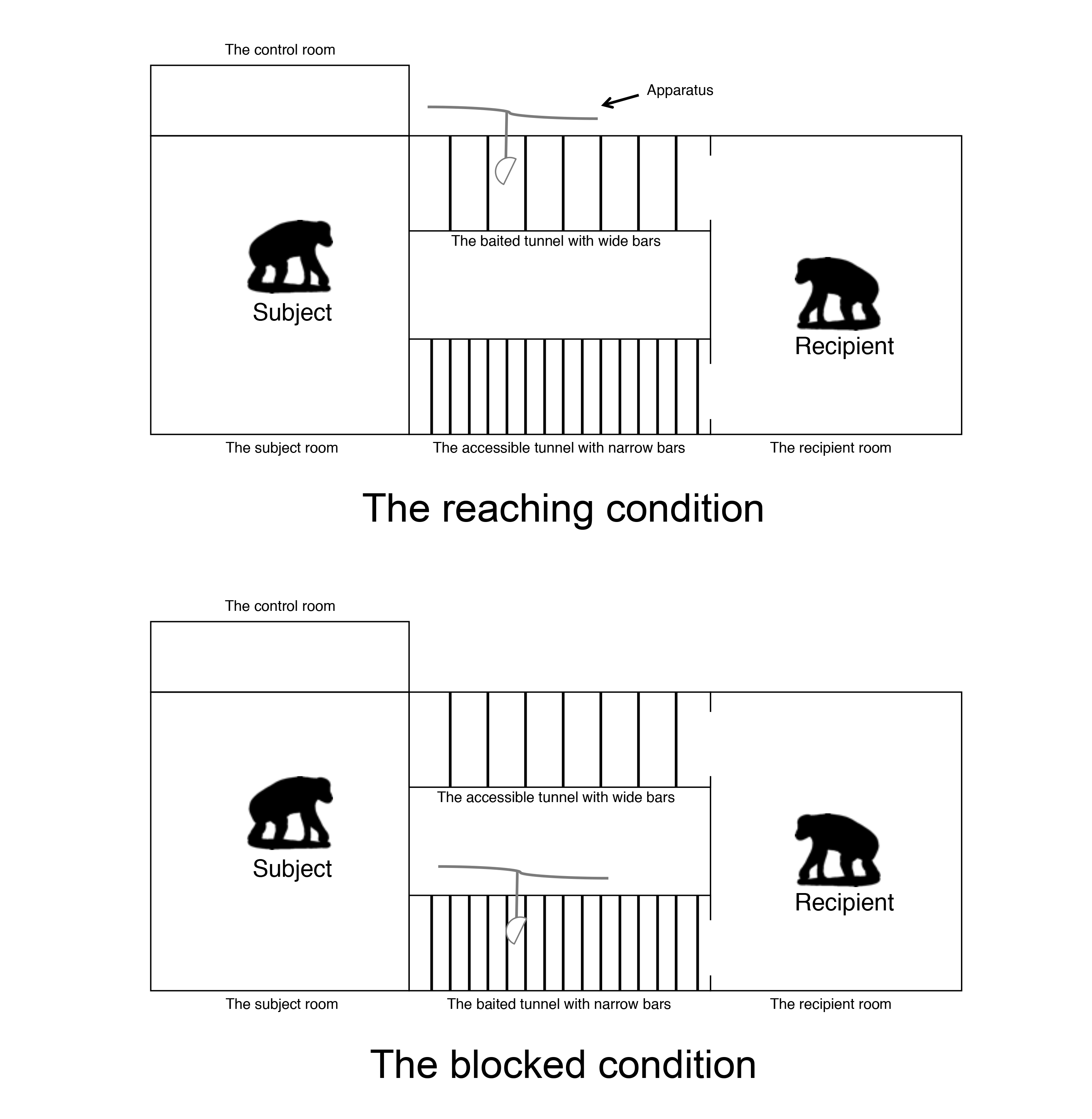
**

**Fig. S1.** The setup of the reaching and the blocked conditions in experiment 1. Note that both (*A*) and (*B*) depict the experimental condition. In the control condition, the recipient was in the control room.

**
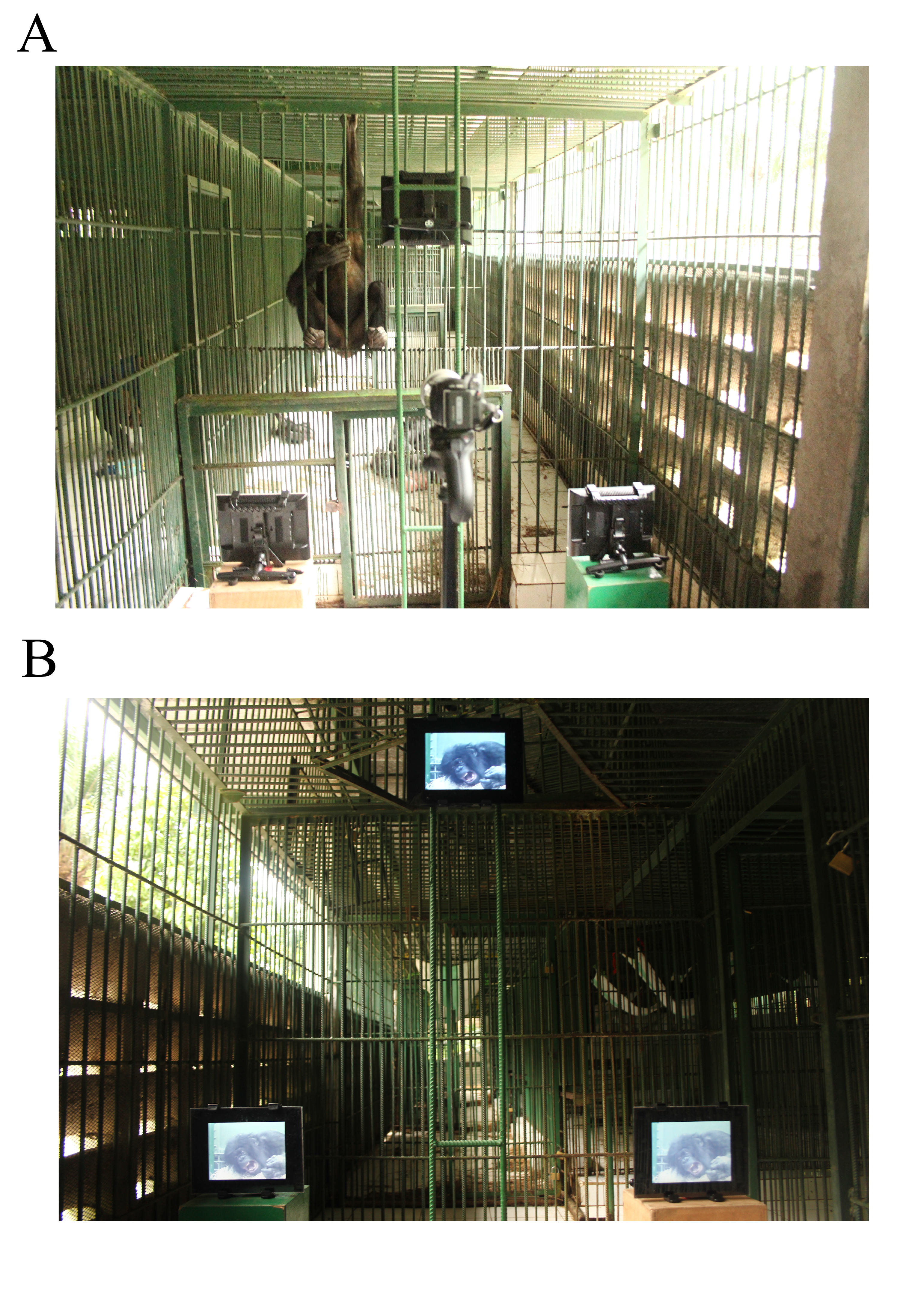
**

**Fig. S2.** The setup of the three screens in experiment 2.

**
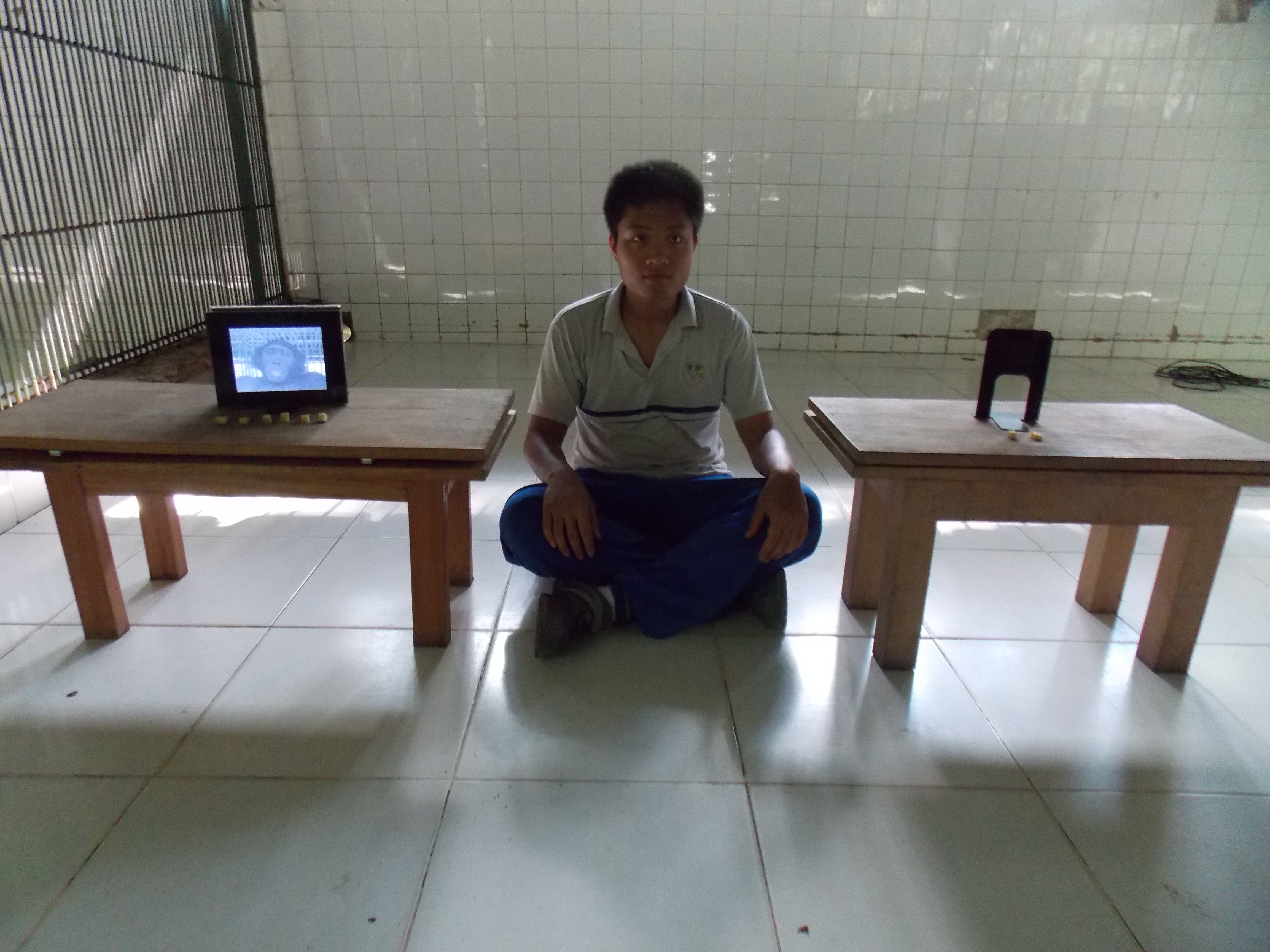
**

**Fig. S3.** The delay option (left) and the instant option (right).
