# Supplementary material for: Bonobos respond prosocially toward members of other groups
Source: Sci Rep. 2017 Nov 7;7:14733. doi: 10.1038/s41598-017-15320-w (PMC5676687; doi:10.1038/s41598-017-15320-w)
Supplement: Supplementary file 2 — Table S1 [file 41598_2017_15320_MOESM2_ESM.doc]

**Table S1.** Subjects of experiment 1.

| Subject | Age | Recipient | Subject-recipient sex combination | Condition | # releases in Exp. | # releases in Control |
| --- | --- | --- | --- | --- | --- | --- |
| Bolomba | 8 | Kasongo | F-M | Reaching | 0 | 0 |
| Dilolo | 11 | Lukuru | M-F | Blocked | 0 | 0 |
| Ilebo | 10 | Lukuru | M-F | Blocked | 7 | 0 |
| Kalina | 15 | Kananga | F-F | Reaching | 1 | 1 |
| Kananga | 5 | Mabali | F-M | Reaching | 0 | 0 |
| Kasongo | 10 | Kipolo | M-M | Blocked | 0 | 0 |
| Katako | 8 | Kipolo | F-M | Blocked | 3 | 0 |
| Kikwit | 15 | Oshwe | M-M | Reaching | 0 | 0 |
| Lisala | 11 | Lukuru | F-F | Blocked | 0 | 0 |
| Lomako | 6 | Mabali | M-M | Reaching | 7 | 1 |
| Lukuru | 7 | Kikwit | F-M | Blocked | 8 | 3 |
| Mabali | 11 | Kipolo | M-M | Blocked | 4 | 1 |
| Malayika | 6 | Lukuru | F-F | Reaching | 0 | 0 |
| Matadi | 12 | Lukuru | M-F | Blocked | 0 | 0 |
| Oshwe | 6 | Katako | M-F | Reaching | 1 | 0 |
| Sandoa | 6 | Malayika | F-F | Reaching | 5 | 2 |
