# Supplementary material for: Bonobos respond prosocially toward members of other groups
Source: Sci Rep. 2017 Nov 7;7:14733. doi: 10.1038/s41598-017-15320-w (PMC5676687; doi:10.1038/s41598-017-15320-w)
Supplement: Supplementary file 3 — Table S2 [file 41598_2017_15320_MOESM3_ESM.doc]

**Table S2.** Subjects of experiment 2.

| Subject | Sex | Age | Alone? | # yawns in Stranger-Yawn condition | # yawns in Stranger-Control condition | # yawns in Groupmate-Yawn condition | # yawns in Groupmate-Control condition |
| --- | --- | --- | --- | --- | --- | --- | --- |
| Api | M | 11 | Yes | 3 | 1 | 0 | 0 |
| Bili | M | 10 | Yes | 4 | 3 | 0 | 2 |
| Bisengo | M | 6 | with Maya | 0 | 0 | 6 | 0 |
| Eleke | M | 6 | Yes | 1 | 0 | 4 | 0 |
| Elikya | F | 6 | Yes | 2 | 4 | 0 | 0 |
| Ilebo | M | 9 | Yes | 2 | 0 | 4 | 5 |
| Isiro | F | 13 | with Likasi | 5 | 3 | 1 | 0 |
| Kalina | F | 13 | with Malayika | 0 | 0 | 3 | 0 |
| Kananga | F | 3 | Yes | 0 | 0 | NA | NA |
| Kasongo | M | 9 | Yes | NA | NA | 3 | 0 |
| Kikwit | M | 13 | Yes | 0 | 0 | 1 | 0 |
| Kisantu | F | 12 | with newborn infant | 8 | 5 | 2 | 4 |
| Likasi | F | 10 | Yes | 0 | 0 | 2 | 2 |
| Lisala | F | 10 | Yes | 3 | 0 | NA | NA |
| Lomami | M | 12 | Yes | 3 | 1 | 0 | 0 |
| Lukuru | F | 5 | Yes | 0 | 0 | NA | NA |
| Mabali | M | 8 | Yes | 1 | 4 | 0 | 3 |
| Malayika | F | 4 | with Kalina | 3 | 1 | 0 | 0 |
| Manono | M | 17 | Yes | 0 | 1 | 0 | 0 |
| Maya | F | 18 | with Bisengo | 1 | 0 | 1 | 0 |
| Mbandaka | M | 9 | Yes | 7 | 4 | 5 | 3 |
| Sake | F | 6 | Yes | 1 | 0 | 12 | 2 |
| Semendwa | F | 14 | with newborn infant | 4 | 2 | 5 | 7 |
| Waka | F | 5 | Yes | 0 | 0 | 0 | 0 |
| Yolo | M | 7 | Yes | 0 | 0 | 0 | 1 |
