# Supplementary material for: Bonobos respond prosocially toward members of other groups
Source: Sci Rep. 2017 Nov 7;7:14733. doi: 10.1038/s41598-017-15320-w (PMC5676687; doi:10.1038/s41598-017-15320-w)
Supplement: Supplementary file 4 — Table S3 [file 41598_2017_15320_MOESM4_ESM.doc]

**Table S3.** Subjects of experiment 3.

| Subject | Sex | Age | Tested in exp. 2? | Day 1 condition | Day 1, # choices of the delay option | Day 2 condition | Day 2, # choices of the delay option |
| --- | --- | --- | --- | --- | --- | --- | --- |
| Api | M | 12 | Yes | Stranger | 8 | Groupmate | 7 |
| Bandundu | F | 15 | No | Stranger | 6 | Groupmate | 5 |
| Bili | M | 10 | Yes | Groupmate | 3 | Stranger | 5 |
| Dilolo | M | 11 | No | Groupmate | 4 | Stranger | 3 |
| Eleke | M | 7 | Yes | Stranger | 5 | Groupmate | 5 |
| Fizi | M | 12 | No | Groupmate | 5 | Stranger | 5 |
| Ilebo | M | 10 | Yes | Stranger | 6 | Groupmate | 5 |
| Kalina | F | 14 | Yes | Groupmate | 6 | Stranger | 3 |
| Kasongo | M | 9 | Yes | Groupmate | 5 | Stranger | 7 |
| Katako | F | 7 | No | Groupmate | 5 | Stranger | 7 |
| Kikwit | M | 13 | Yes | Stranger | 8 | Groupmate | 5 |
| Kisantu | F | 13 | Yes | Stranger | 5 | Groupmate | 5 |
| Mabali | M | 8 | Yes | Stranger | 8 | Groupmate | 7 |
| Mbandaka | M | 10 | Yes | Groupmate | 5 | NA | NA |
| Malayika | F | 5 | Yes | Groupmate | 6 | Stranger | 5 |
| Masisi | F | 5 | No | Stranger | 6 | Groupmate | 6 |
| Matadi | M | 11 | No | Groupmate | 5 | NA | NA |
| Muanda | F | 8 | No | Groupmate | 6 | Stranger | 6 |
| Opala | F | 17 | No | Stranger | 5 | Groupmate | 6 |
| Sake | F | 7 | Yes | Groupmate | 7 | Stranger | 7 |
| Waka | F | 6 | Yes | Stranger | 8 | Groupmate | 7 |
| Yolo | M | 8 | Yes | Groupmate | 6 | Stranger | 8 |
